# Effects of Angiotensin-Converting Enzyme Inhibition on the Recurrence and Internal Structure of Chronic Subdural Hematomas

**DOI:** 10.3390/jcm13164591

**Published:** 2024-08-06

**Authors:** Michael Veldeman, Hani Ridwan, Mohamed Alzaiyani, Rastislav Pjontek, Benedikt Kremer, Anke Hoellig, Hans Clusmann, Hussam Hamou

**Affiliations:** 1 Department of Neurosurgery, RWTH Aachen University Hospital, Pauwelsstrasse 30, 52074 Aachen, Germany; 2 Department of Diagnostic and Interventional Neuroradiology, RWTH Aachen University, 52062 Aachen, Germany

**Keywords:** chronic subdural hematoma, angiotensin-converting enzyme inhibitor, burr hole craniotomy

## Abstract

**Background/Objectives:** Chronic subdural hematoma (cSDH) is a common disease of growing significance due to the increasing use of antithrombotic drugs and population aging. There exists conflicting observational evidence that previous treatment with angiotensin-converting enzyme (ACE) inhibitors reduces the rate of cSDH recurrence. This study assesses the hypothesis that ACE inhibitors may affect recurrence rates by altering hematoma membrane formation. **Methods:** All patients with chronic subdural hematoma who were operated upon in a single university hospital between 2015 and 2020 were considered for inclusion. Hematomas were classified according to their structural appearance in computed tomography (CT) imaging into one of eight subtypes. Patients’ own medication, prior to hospitalization for cSDH treatment, was noted, and the use of ACI-inhibitors was identified. **Results:** Of the included 398 patients, 142 (35.9%) were treated with ACE inhibitors before admission for cSDH treatment. Of these, 115 patients (81.0%) received ramipril, 13 received patients lisinopril (11.3%), and 11 patients (9.6%) received enalapril. Reflecting cardiovascular comorbidity, patients on ACE inhibitors were more often simultaneously treated with antithrombotics (63.4% vs. 42.6%; *p* ≤ 0.001). Hematomas with homogenous hypodense (OR 11.739, 95%CI 2.570 to 53.612; *p* = 0.001), homogenous isodense (OR 12.204, 95%CI 2.669 to 55.798; *p* < 0.001), and homogenous hyperdense (OR 9.472, 95%CI 1.718 to 52.217; *p* < 0.001) architectures, as well as the prior use of ACE inhibitors (OR 2.026, 95%CI 1.214 to 3.384; *p* = 0.007), were independently associated with cSDH recurrence. **Conclusions:** Once corrected for hematoma architecture, type of surgery, and use of antithrombotic medication, preoperative use of ACE inhibitors was associated with a twofold increase in the likelihood of hematoma recurrence.

## 1. Introduction

Chronic subdural hematoma (cSDH) is a relatively common and increasingly significant intracranial disease entity. Taking into account a broad geographical and age-related variability, the overall incidence is estimated to be around 8 individuals per 100,000 per year [1]. Due to population aging and the increased prescription of antithrombotic drugs [2], the incidence of cSDH is expected to steadily rise, and along with it, its socioeconomic burden [3,4]. The exact pathophysiological processes underlying cSDH remain unclear. However, it is generally presumed that a minor acute subdural hemorrhage is the initial initiator of an inflammatory process, which eventually leads to the collection of a subdural fluid collection growing in size [5]. During this delayed development, vascularized membranes form, and recurrent bleeding and fluid extravasation originating from these neomembranes are considered to be responsible for the volume expansion seen in cSDH [6].

Treatment of space-occupying symptomatic hematomas generally consists of surgical evacuation, as it can achieve swift symptom relief. Although treatment practices vary considerably, burr hole craniotomy (BHC) with the placement of one or more subdural drains is commonly performed [7]. Surgical treatment is still complicated by high hematoma recurrence rates, leading to the necessity of retreatment and rising costs. To date, no effective non-surgical treatments exist to prevent or treat cSDH [3].

In a retrospective cohort analysis of 438 patients treated surgically for cSDH, the recurrence rate proved to be lower in patients with prior antihypertensive treatment by means of an angiotensin-converting enzyme (ACE) inhibitor [8]. This effect became stronger the longer patients had been previously treated with ACE inhibitors. The authors hypothesized that this was mediated by the decrease in production of vascular endothelial growth factor (VEGF) induced by this medication. This was corroborated by a lower concentration of VEGF in the hematoma fluid in a subgroup of this cohort. In contrast, Neidert et al. observed larger initial hematoma volumes and a higher recurrence rate in elderly patients on ACE inhibitors [9]. In a similar comparison between patients with cSDH with or without previous ACE inhibitor treatment, Bartek et al. identified no difference in the recurrence of cSDH [10].

In a recent analysis of 408 patients treated for cSDH, our group identified internal hematoma architecture on computed tomography (CT) imaging to be associated with postoperative recurrence [11]. Hematoma organization with the development of neomembranes resulted in fewer postoperative recurrences, whereas hematomas with sediment formation, indicative of coagulopathy, presented with the highest likelihood of recurrence. Here, we concluded that fibroproliferation leading to membranization of the hematoma and development of septa could be an attempt towards natural repair.

The primary goal of this study was to assess whether prior intake of ACE inhibitors affected the hematoma’s architecture and formation of membranes on CT imaging. The hypothesis is that suppression of angiogenesis might hamper fibroproliferation and results in less pronounced radiological membranization. This might provide a causal link concerning how ACI inhibitors may affect cSDH recurrence. In addition, we aim to pool our results with those of previous similar studies in respect to the recurrence of cSDH in patients with or without prior ACE inhibitor treatment.

## 2. Materials and Methods

### 2.1. Study Population and Design

All patients with cSDH who were operated upon in a single university hospital (RWTH Aachen University Hospital, Aachen, Germany) between 2015 and 2020 were considered for inclusion. A smaller subgroup of this collective, focusing on hematoma architecture, has been previously analyzed, and the results published [11,12]. The current study was approved by the ethical review board of the RWTH Aachen University Hospital (EK399/20) and was registered in the German Clinical Trials Register (DRKS00025280). This text is drafted in accordance with the Strengthening the Reporting of Observational studies in Epidemiology (STROBE) statement for reporting observational studies.

Patients were excluded (1) if initial CT imaging was missing (patient referred to us with images that were not uploaded to our picture archiving and communication system, (2) in case of underlying intracranial hypotension leading to cSDH, or (3) after cranial procedures contributing to cSDH formation. All surgically treated patients were included, irrespective of the type of (initial) surgery. Data were collected retrospectively by screening digital hospital records, with the extraction of demographic data, medical history including medication, presenting clinical symptoms, pre- and postoperative hematoma characteristics, and hematoma recurrence.

### 2.2. Treatment Algorithm

We previously published our institutional cSDH treatment algorithm and provide a short summary here [11]. Surgery was considered for patients with cSDH presenting with a neurological deficit, i.e., a speech disorder, gait imbalance, or hemiparesis. Asymptomatic cSDH was treated when imaging demonstrated clear mass effect in the form of midline shift, compression of the ventricular system, and/or sulcal effacement. Burr hole craniotomy with placement of one or two non-suction subdural silicon drains was the primary treatment of choice. Twist-drill craniostomy in local anesthesia was reserved for homogenous hematomas in patients with severe comorbidity. Mini-craniotomy was only considered when incomplete removal was anticipated due to the presence of hyperdense clots on CT imaging. The choice of which surgical procedure to perform was left to the surgeon’s discretion. Apart from drainage removal on day two or three in patients with burr hole craniotomy, no differences in postoperative care existed between different treatment regimens. Along with other anti-hypertensive drugs, intake of ACE inhibitors was not interrupted preoperatively.

Patients were either discharged with full symptom relief or postoperative imaging demonstrating the resolution of the space-occupying effect of the hematoma. Follow-up imaging was routinely performed 14 to 21 days after treatment and continued in 2 to 3 week intervals until radiological remission. Recurrence was defined as an increase in the volume of a residual or newly formed hematoma with mass effect (as defined by the criteria above) or the new development or reappearance of neurological symptoms leading to the need for redo surgery. Treatment of recurrence consisted of a burr hole craniotomy with the placement of subdural silicon drains.

### 2.3. Radiological Evaluation

**Figure 1:**
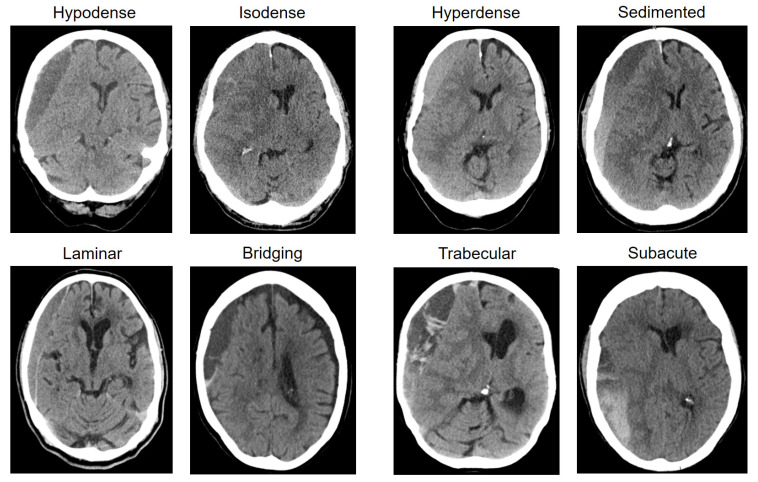
Eight chronic subdural hematoma types based on the appearance of internal architecture in computed tomography imaging (adapted from Hamou et al. [11]).

Preoperative hematoma size was measured in CT imaging (axial only) as the length along the longest axis (mm) and width at its widest (mm). In addition, volume (mL) was reconstructed with the assistance of software (Brainlab, Munich, Germany). The presence of midline shift was noted and measured (mm). Subsequently, hematomas were classified based on their structural appearance in CT imaging into one of eight subtypes by two independent assessors (HH and MV), as described previously [11]: (1) homogenous hypodense, consisting of a homogenous hematoma appearing hypodense compared to brain tissue; (2) homogenous isodense, consisting of a homogenous hematoma isodense compared to brain tissue; (3) homogenous hyperdense, consisting of a homogenous hematoma hyperdense in relation to brain tissue with a lack of an immediate history of head trauma; (4) sedimented, hemoglobin sediments are separated from less dense content by gravity during imaging; (5) laminar, a hyperdense visceral or parietal membrane is visible; (6) bridging, a countable number of internal membranes connects the visceral and parietal membrane; (7) trabecular type, a more complex and diffuse membrane structure precluding counting of membranes; (8) subacute, chronic subdural hematoma with small acute blood components with or without visceral/parietal membranes and a lack of an immediate history of head trauma [11]. An exemplary image of each hematoma types is depicted in Figure 1 (adapted from [11]).

### 2.4. Prior Treatment with ACE Inhibitors and Outcomes

Patients’ own medication prior to admission to the hospital was noted and the use of ACI-inhibitors was identified.

Clinical outcome was extracted from the last available documented follow-up visit, categorized according to the GOS, and dichotomized into a favorable (GOS4–5) or unfavorable (GOS1–3) outcome.

### 2.5. Statistical Analysis

Continuous data are presented as the mean and standard deviation if normally distributed, and as median and interquartile range (Q_1_ to Q_3_) for non-normally distributed variables. Categorical data are summarized as proportions. After normality testing via the Shapiro–Wilk test and plotting of data, the appropriate statistical test was selected. Categorical data were tested with the χ^2^-test, and continuous data were selected by means of the unpaired *t*-test or Mann–Whitney U test. A binary logistic regression model was constructed, adding factors with univariate *p*-values < 0.10 in a single block [13], or based on clinical relevance. Associations in the regression analysis are denoted as odds ratios (OR) with 95% confidence intervals (CI). Before inclusion, variables were tested for outliers via plotting, and multicollinearity was evaluated via the assessment of the variance inflation factor with a cutoff of 2.5. All statistical analyses were performed using IBM SPSS Statistics 25 (SPSS Inc., Chicago, IL, USA). Statistical significance was defined as a two-sided *p* < 0.05.

## 3. Results

**Figure 2:**
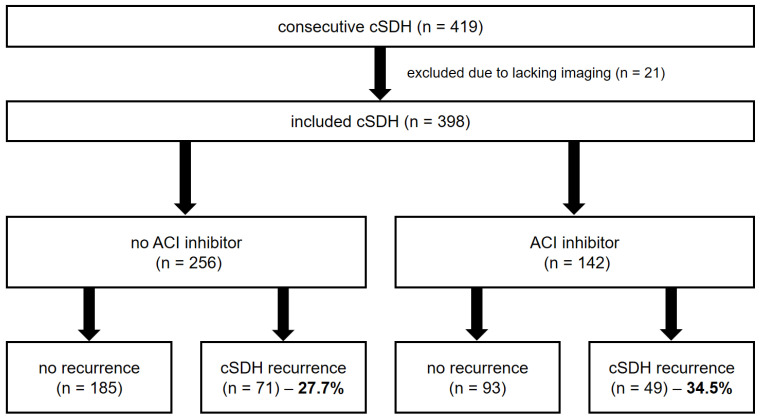
Flowchart of patients’ inclusion. ACI, angiotensin-converting enzyme; cSDH, chronic subdural hematoma.

In total, 419 consecutive cSDH patients were treated at our institution during the inclusion time window. Based on the lack of initial CT imaging, 21 patients were excluded. Figure 1 depicts the inclusion process. Of the 398 patients included, 142 (35.7%) were treated with ACE inhibitors prior to admission for cSDH. Of these, 115 patients (81.0%) received ramipril, 13 patients received lisinopril (11.3%), 11 patients (9.6%) received enalapril, and there was a single patient on each one of the following ACE inhibitors: captopril, quinapril, and perindopril. The inclusion process is depicted as a flowchart (Figure 2). 

Of patients previously treated with ACE inhibitors, 34.5% (n = 49) developed recurrence (*p* = 0.159). This proved comparable with the remaining 256 patients without prior ACE inhibitor treatment, of which 71 (27.7%; *p* = 0.159) developed recurrence. Subgroups of patients with or without previous ACE inhibitor treatment differed in relevant baseline characteristics. Apart from the obvious discrepancy in the prevalence of arterial hypertension, patients with previous ACE inhibitor treatment were older (73.8 ± 12.6 vs. 77.1 ± 11.2; *p* = 0.008) and more commonly of the male sex (73.2% vs. 60.5%; *p* = 0.011) (see Table 1). In addition, reflecting accompanying cardiovascular comorbidity, patients on ACE inhibitors were more often simultaneously treated with antithrombotic medication (63.4% vs. 42.6%; *p* ≤ 0.001).

Comparing the size of hematomas in patients with or without prior ACE inhibitor treatment, neither width and length nor volume differed between these subgroups (Table 2). Looking at internal architecture, no differences were observed in the proportions of patients with membranous hematomas between subgroups. A comparable proportion of patients in the group with ACE inhibitor treatment reached a favorable outcome (118 (84.9%) vs. 227 patients (89.4%); *p* = 0.982). Clinical outcome was assessed after a median of 82.5 days [37 to 161].

A logistic regression model was built to identify factors associated with hematoma recurrence. Based on univariate analysis, internal architecture (eight types), type of surgery (three types), and use of antithrombotic medication were identified to be included into the model. Additionally, prior intake of ACE inhibitors was added as a final factor. The logistic regression model proved significant as χ^2^(10) = 68.549, *p* < 0.001. The model explained 22.4% (Nagelkerke R^2^) of the variance in recurrence and classified 74.1% of cases correctly. Of the predictor variables, hematomas with a homogenous hypodense (OR 11.739, 95%CI 2.570 to 53.612; *p* = 0.001), homogenous isodense (OR 12.204, 95%CI 2.669 to 55.798; *p* < 0.001), and homogenous hyperdense (OR 9.472, 95%CI 1.718 to 52.217; *p* < 0.001) architecture, as well as the prior use of ACE inhibitors (OR 2.026, 95%CI 1.214 to 3.384; *p* = 0.007), were associated with the recurrence of cSDH (see Table 3).

### Pooled Results

**Figure 3:**
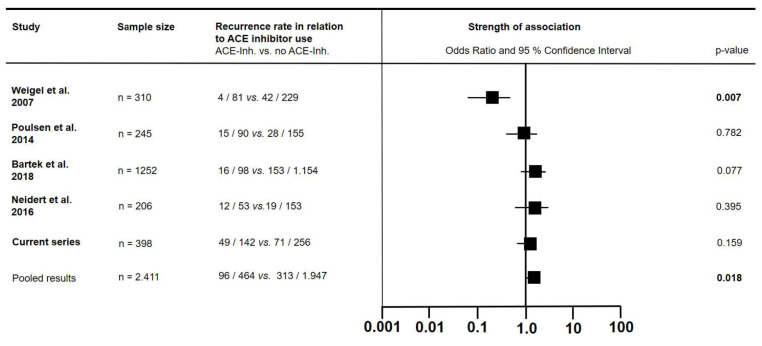
The literature overview of factors associated with the recurrence of chronic subdural hematoma in patients with or without prior treatment with angiotensin-converting enzyme inhibitors. *p*-values below the preset α-level of 0.05 are marked in bold [8,9,10,14].

The results of all four known trials assessing the recurrence of cSDH in patients with or without previous antihypertensive treatment with ACE inhibitors were combined [8,9,10,14]. In this pooled cohort of 2.411 patients, 96 (20.7%) out of 464 patients on ACE inhibitors developed recurrent hematoma vs. 313 (16.1%) patients out of a total of 1.947 without prior ACE inhibitor treatment (OR 1.362, 95% CI 1.055 to 1.758; *p* = 0.018) (see Figure 3).

## 4. Discussion

We previously demonstrated lower cSDH recurrence rates in hematomas with a membranous architecture on CT imaging [11]. In the current study, we hypothesized that ACE inhibitors might affect the formation of hematoma membranes. In our assessed cohort, patients receiving hypertensive treatment with ACE inhibitors were more likely to receive concomitant antithrombotic drugs. Once corrected for hematoma architecture, type of surgery, and use of antithrombotic medication, in a multivariate regression model, preoperative use of ACE inhibitors was associated with a twofold increase in the likelihood of hematoma recurrence. As to what could cause this effect, it cannot be excluded that ACE inhibitor use is reflective of a more severe underlying comorbidity and is, by itself, not directly causally related to cSDH recurrence. In addition, the distribution of architectural hematoma subtypes did not differ in relation to ACE inhibitor use.

Although the surgical strategy for cSDH is usually straightforward, treatment is still frequently complicated by recurrence and need for reoperation. Recurrence is affected by a multitude of factors which can be divided into unmodifiable patient-specific characteristics including age-related brain atrophy and a lack of brain compliance versus potentially modifiable factors, such as inflammation and coagulopathy [15]. In a randomized controlled trial, anti-inflammatory treatment with oral dexamethasone as an adjunct to surgery was not able to improve clinical outcome after surgical treatment for cSDH [16].

In a promising 2007 study, preoperative treatment of hypertension with ACE inhibitors yielded a lower recurrence rate in patients with cSDH [8]. Two similarly designed confirmation studies demonstrated opposing results with increased hematoma volume and a higher risk of recurrence in one study [9] versus no effect on recurrence in preoperative ACE inhibitor users in the other [10]. In the first of these studies, patients with coagulopathies were excluded, and in the latter, analyses were not corrected for the intake of antithrombotic drugs. In a prospective double-blind randomized trial, the ACE inhibitor perindopril was compared against a placebo as an adjuvant treatment after surgical evacuation of cSDH [14]. None of the 47 included patients developed hematoma recurrence, making the results difficult to interpret. The authors included a retrospective cohort of cSDH patients and assessed the effect of prior ACE inhibitor treatment on recurrence, with negative results. We have pooled the data of all four studies in combination with data from our current series. The larger cohort size of the study by Bartek et al. [10] counteracted the positive effect of the initial 2007 study [8], resulting in a 36% risk increase for recurrence in ACE inhibitor users in the combined cohort of over 2,000 patients.

Vascular endothelial growth factor (VEGF) is an angiogenic factor which plays a role in many physiological processes, including wound healing, bone formation and hematopoiesis, oxygen homeostasis, neurogenesis, and many more [17]. By promoting vascularization, VEGF can contribute to the restoration of blood supply in response to hypoxia [18,19]. The signaling protein is able to increase vascular permeability through several mechanisms, including the disruption of endothelial junctions, production of nitrous oxide, cytoskeletal changes, and the formation of fenestrations [20]. As such, VEGF can contribute to edema in certain conditions, like inflammation, tumor growth, and wet age-related macular degeneration [21]. Anti-VEGF treatments in the form of antibodies, like aflibercept, bevacizumab, and ranibizumab, among others, have mainly found their application in either the systemic treatment of cancer or the focal treatment of macular degeneration [22,23]. Angiotensin II has been shown to upregulate VEGF expression through various signaling pathways. ACE inhibitors block the conversion of angiotensin I to angiotensin II and thereby decrease the expression of VEGF [24]. It is hypnotized that VEGF plays a role in the formation of membranes and septa in cSDH, and VEGF-induced hyperpermeability might contribute to cSDH growth [25].

Weigel et al. [8] demonstrated lower levels of VEGF in the hematoma fluid of patients previously treated with ACE inhibitors, which was accompanied with lower recurrence rates. In addition, higher levels of VEGF expression were found in cells of the hematoma fluid compared to the hematoma’s membrane, suggesting how the hematoma fluid itself is a stronger promoter of angiogenesis and hyperpermeability [26]. In contrast, Takei et al. observed higher VEGF levels in more organized (trabecular) hematomas, which were associated with lower recurrence rates [27].

Assuming that ACE inhibitors suppress VEGF expression and thereby reduce angiogenesis, one would expect lower levels of hematoma membranization in patients treated with ACE inhibitors, which was not encountered in our series. Overall, the link between ACE inhibitors and VEGF expression is not yet clearly understood, and in other settings, ACE inhibition can mediate improved neovascularization rather than suppression [28].

All previous literature focused on home medication prior to cSDH diagnosis. If this group of medication is suspected to offer prophylactic benefits in respect to cSDH, a population-wide assessment of cSDH incidence in ACE inhibitors users and people without this treatment seems a better study design. The only available randomized data on postoperative use of ACE inhibitors, applied for the sole purpose of reducing hematoma recurrence and not treatment of hypertension, was probably too small in size to yield meaningful results [14]. Based on current insights, there is no evidence that ACE inhibitors are beneficial in cSDH patients, and they might even contribute to recurrence.

## 5. Limitations

This analysis is limited by its retrospective design, exposing data collection to selection and reporting bias. Although our patient catchment area is geographically clearly delineated, hematoma recurrence may have been missed if patients were not re-referred to our center. Patients on ACE inhibitors were more likely to concomitantly receive antithrombotic treatment. Although we statistically corrected for the effects of antithrombotic medication on hematoma recurrence, this difference cannot be neglected. As has been performed in previous similar studies, neither the hematoma fluid nor blood samples were assessed for vascular endothelial growth factor [8]. The retrospective nature of this study impedes such post hoc analysis. This study, therefore, failed to provide any pathophysiological basis on how ACI inhibitors might affect cSDH formation and recurrence. Assessment of the levels of angiogenetic factors, such as vascular endothelial or placenta growth factor in both serum and hematoma, could shed light on the pathophysiological basis by which cSDH formation and healing may be altered by ACE inhibitor use. In this study, although the use of other prior medication (i.e., other antihypertensive drugs) was noted, their effects were not assessed. Also, information on the time point of minor head injury was not available. Therefore, the effect the delay between trauma and surgery might have had on hematoma recurrence, could not be meaningfully assessed. In addition, because the clinical outcome was retrospectively collected, this resulted in a high variability in the duration of follow-up. As a result of the assessment of patients after different periods of recovery, clinical outcome can be biased. Finally, information on the duration of previous treatment with ACI inhibitors prior to the diagnosis of cSDH was not available and could, therefore, not be analyzed.

## 6. Conclusions

Once corrected for hematoma architecture, type of surgery, and the use of antithrombotic medication, preoperative use of ACE inhibitors was associated with a twofold increase in the likelihood of developing hematoma recurrence. The distribution of architectural hematoma subtypes was not affected by prior use of ACE inhibitors.

## Figures and Tables

**Table 1:** Patient-specific baseline characteristics in chronic subdural hematoma, compared between patients with or without previous intake of ACE inhibitors. ACE, angiotensin-converting enzyme; activated; GCS, Glasgow come scale; INR, international normalized ratio; nl, nanoliter; NOAC, new oral anticoagulants; mm, millimeter; PTT, partial thromboplastin time; s, seconds; SD, standard deviation. Q_1_, first quartile; Q_3_, third quartile; *p*-values below the preset α-level of 0.05 are marked in bold.

Demographics—Patient Characteristics	All (n = 398)	No ACE Inhibitor (n = 256)	ACE Inhibitor (n = 142)	*p*-Value
Age—mean ± SD	74.9 ± 77.9	73.8 ± 12.6	77.1 ± 11.2	**0.008**
Sex—female (%)/male (%)	139 (34.9)/259 (65.1)	101 (39.5)/155 (60.5)	38 (26.8)/104 (73.2)	**0.011**
**Initial presentation**				
Initial GCS—median [Q_1_ to Q_3_]	15 [14 to 15]	15 [15 to 15]	15 [14 to 15]	0.409
Markwalder grading—median [Q_1_ to Q_3_]	2 [1 to 2]	2 [1 to 2]	2 [1 to 2]	0.297
Documented trauma—no. (%)	274 (68.8)	177 (69.1)	97 (68.3)	0.864
Preoperative deficit—no. (%)	366 (92.6)	233 (92.5)	133 (93.7)	0.656
Phatic disorder	91 (22.9)	56 (21.9)	35 (24.6)	0.583
Paresis	205 (51.5)	125 (48.8)	80 (56.3)	0.199
Gait disturbance	163 (41.0)	102 (39.8)	61 (43.0)	0.654
Personality changes/confusion	20 (5.0)	16 (6.3)	4 (2.8)	0.125
**Comorbidity—no. (%)**				
Hypertension	214 (53.8)	72 (28.1)	142 (100)	**<0.001**
Atrial fibrillation	99 (24.9)	60 (23.4)	39 (27.5)	0.373
Coronary arterial disease	134 (33.7)	72 (28.1)	62 (43.7)	0.002
Diabetes	76 (19.1)	43 (16.8)	33 (23.2)	0.117
Cancerous disease	62 (15.6)	44 (17.2)	18 (12.7)	0.234
Alcohol abuse	20 (5.0)	13 (5.1)	7 (4.9)	0.948
**Prior Medication—no. (%)**				
Antithrombotics (all)	199 (50.0)	109 (42.6)	90 (63.4)	**<0.001**
Antiplatelet	112 (28.2)	62 (24.2)	50 (35.5)	0.017
Anticoagulants	54 (13.6)	26 (10.2)	28 (20.0)	0.060
Of which NOAC	20 (5.1)	12 (4.7)	8 (5.8)	0.638
**Coagulation tests**				
Preoperative PTT (s)	29.5 ± 13.9	27.8 ± 17.0	29.1 ± 4.4	0.621
Preoperative INR	1.4 ± 4.6	1.2 ± 0.6	1.9 ± 7.6	0.138
Preoperative platelet count (/nl)	241.7 ± 87.0	242.2 ± 83.4	240.0 ± 93.3	0.876

**Table 2:** Hematoma-specific baseline characteristics and clinical outcome in patients with chronic subdural hematoma subdivided into patients with or without prior intake of ACE inhibitors. Membranous hematomas include laminar, bridging, and trabecular. ACE, angiotensin-converting enzyme; DVT, deep-vein thrombosis; GOS, Glasgow outcome scale; mm, millimeter; SD, standard deviation. *p*-values below the preset α-level of 0.05 are marked in bold.

Hematoma Characteristics and Outcome	All (n = 398)	No ACE Inhibitor (n = 256)	ACE Inhibitor (n = 142)	*p*-Value
**Hematoma imaging characteristics**				
Left side—no. (%)	133 (37.5)	82 (35.7)	51 (40.8)	0.598
Bilateral—no. (%)	97 (24.4)	61 (23.8)	36 (25.4)	0.734
Width (mm)—mean ± SD	21.5 ± 6.1	21.3 ± 5.8	21.7 ± 6.6	0.433
Length (mm)—mean ± SD	121.9 ± 48.2	120.9 ± 58.1	123.9 ± 20.5	0.553
Volume (mL)—mean ± SD	150.1 ± 118.3	146.2 ± 137.5	157.0 ± 71.7	0.386
Midline shift—no. (%)	274 (68.8)	175 (68.4)	99 (69.7)	0.738
MLS (n = 274) (mm)—mean ± SD	9.3 ± 4.2	9.1 ± 4.2	9.6 ± 4.1	0.276
**Internal architecture—no. (%)**				0.730
Homogenous hypodense	81 (20.4)	51 (19.9)	30 (21.1)	
Homogenous isodense	86 (21.6)	62 (24.2)	24 (16.9)	
Homogenous hyperdense	22 (5.5)	16 (6.3)	6 (4.2)	
Sedimented	27 (6.8)	16 (6.3)	11 (7.7)	
Laminar	51 (12.8)	32 (12.5)	19 (13.4)	
Bridging	38 (9.5)	22 (8.6)	16 (11.3)	
Trabecular	67 (16.8)	41 (16.0)	26 (18.3)	
Subacute	26 (6.5)	16 (6.3)	10 (7.0)	
Membranous hematoma	152 (38.2)	93 (36.3)	59 (41.5)	0.304
**Surgical treatment—no. (%)**				0.153
Burr hole	270 (67.8)	169 (66.0)	101 (71.1)	
Twist drill	117 (29.4)	82 (32.0)	35 (24.6)	
Bone flap	11 (2.8)	5 (2.0)	6 (4.2)	
**Postoperative outcome**				
GOS—last clinical check-up—no. (%)				0.075
Good recovery	290 (73.6)	196 (76.9)	94 (67.6)	
Moderate disability	54 (13.7)	32 (12.5)	22 (15.8)	
Severe disability	29 (7.4)	13 (5.1)	16 (11.5)	
Vegetative state	3 (0.8)	1 (0.4)	2 (1.4)	
Dead	18 (4.6)	13 (5.1)	5 (3.6)	
Missing outcome	4 (1.0)	n/a	n/a	
Favorable outcome	345 (87.8)	227 (89.4)	118 (84.9)	0.195
Length of follow-up (days)—median [Q_1_ to Q_3_]	82.5 [37 to 161]	82 [38 to 158]	86 [33 to 167]	0.982
**Recurrence—no. (%)**	120 (30.2)	71 (27.7)	49 (34.5)	0.158

**Table 3:** Hematoma-specific baseline characteristics and clinical outcome in patients with chronic subdural hematoma subdivided into patients with or without prior intake of ACE inhibitors. ^‡^, Reference category. ^§^, multivariate α-level of 0.025 (Bonferroni correction). *p*-values below the preset α-level of 0.05 or 0.025 are marked in bold. ACE, angiotensin-converting enzyme; mL, milliliter; SD, standard deviation.

Contribution to Recurrence	All (n = 398)	No Recurrence (n = 278)	Recurrence (n = 120)	Univariate *p*-Value	Adjusted Odds Ratio	95% Confidence Interval	Multivariate *p*-Value ^§^
**Patient characteristics**							
Age—mean ± SD	74.9 ± 77.9	75.3 ± 12.3	74.0 ± 11.1	0.311			
Sex—female (%)/male (%)	139 (34.9)/259 (65.1)	98 (35.3)/180 (64.7)	41 (34.2)/79 (65.8)	0.835			
Documented trauma—no. (%)	274 (68.8)	196 (70.5)	78 (65.0)	0.277			
Preoperative deficit—no. (%)	366 (92.9)	257 (93.5)	109 (91.6)	0.511			
Alcohol abuse	20 (5.0)	13 (4.7)	7 (5.8)	0.628			
Antitrombotics (all)	199 (50.0)	150 (54.0)	49 (40.8)	0.016 ^†^	0.571	0.348 to 0.938	0.027
Volume (mL)—mean ± SD	150.1 ± 118.3	146.1 ± 134.3	159.2 ± 67.8	0.345			
**Hematoma characteristics and treatment**							
**Internal Architecture—no. (%)** *				**<0.001** ^†^			
Homogenous hypodense	81 (20.4)	45 (55.6)	36 (44.4)		11.739	2.570 to 53.612	**0.001**
Homogenous isodense	86 (21.6)	52 (60.5)	34 (39.5)		12.204	2.669 to 55.798	**0.001**
Homogenous hyperdense	22 (5.5)	14 (63.6)	8 (36.4)		9.472	1.718 to 52.217	**0.010**
Sedimented	27 (6.8)	13 (48.1)	14 (51.9)		17.415 ^‡^	3.353 to 90.459	**<0.001**
Laminar	51 (12.8)	43 (84.3)	8 (15.7)		3.322	0.645 to 17.118	0.151
Bridging	38 (9.5)	36 (94.7)	2 (5.3)		^‡^		
Trabecular	67 (16.8)	52 (77.6)	15 (22.4)		5.896	1.235 to 28.152	0.026
Subacute	26 (6.5)	23 (88.5)	3 (11.5)		2.636	0.379 to 18.334	0.327
**Surgical treatment—no. (%)** *				**<0.001** ^†^			
Burr hole	270 (67.8)	211 (78.1)	59 (21.9)		0.412	0.074 to 2.303	0.313
Twist drill	117 (29.4)	58 (49.6)	59 (50.4)		1.233	0.227 to 6.695	0.808
Bone flap	11 (2.8)	9 (81.8)	2 (18.2)		^‡^		
ACE Inhibitor use—no. (%)	142 (35.7)	93 (33.5)	49 (40.8)	0.159 ^†^	2.026	1.214 to 3.384	**0.007**

## Data Availability

The raw data on which this study and the analyses are based can be made available to qualified researchers on reasonable request.

## Abbreviations

ACE	Angiotensin-converting enzyme
CI	Confidence interval
cSDH	Chronic subdural hematoma
CT	Computed tomography
GOS	Glasgow outcome scale
mm	Millimeter
mL	Milliliter
n/a	Not available
NOAC	New oral anticoagulants
OR	Odds ratio
Q_1_ to Q_3_	First quartile to third quartile
SD	Standard deviation
VEGF	Vascular endothelial growth factor
